# Combined use of *Beauveria bassiana* and the predatory mite *Neoseiulus californicus* to suppress *Tetranychus urticae* on greenhouse-grown sweet pepper

**DOI:** 10.1007/s10493-026-01190-3

**Published:** 2026-09-01

**Authors:** Miguel Michereff-Filho, Rogerio Biaggioni Lopes, Paloma Alves da Silva, Elisangela Gomes Fidelis, Atos Mendes do Vale

**Affiliations:** 1 Embrapa Vegetables, BR 090 Road km 09, Federal District 70275-970 Brasília, Brazil; 2 Embrapa Genetic Resources and Biotechnology, PqEB W5 North Avenue, Federal District 70770-917 Brasília, Brazil; 3 PPG Agronomy, University of Brasília (UnB), Federal District 70910-000 Brasília, Brazil; 4 PPG Agricultural Sciences - Agronomy, Goiano Federal Institute of Education, Science and Technology (IFGO), Campus Rio Verde, Rio Verde, Goiás State 75901-970 Rio Verde, Brazil

**Keywords:** Spider mite, Phytoseiidae, Acaropathogenic fungus, Biological control

## Abstract

Predatory mites and invertebrate-pathogenic fungi are important biological control tools for managing populations of the two-spotted spider mite, *Tetranychus urticae*, in horticultural field crops. Here, we evaluated strategies for the combined use of a mite-pathogenic strain of *Beauveria bassiana* and *Neoseiulus californicus* to suppress *T. urticae* populations on sweet pepper plants. First, we compared the performance of both biocontrol agents, applied alone or in combination, under greenhouse conditions. A decline in *T. urticae* populations was observed 7 days after the release of the predatory mite (53–62%), the application of fungal conidia (77–81%), and their combined use (73–81%). However, in the combined treatment, *N. californicus* abundance was lower on plants sprayed with *B. bassiana* than on unsprayed plants. We then evaluated a temporal selectivity strategy in lab and greenhouse conditions, releasing the predatory mite at varying intervals after fungal application, to improve *T. urticae* suppression. Intervals of 6 days between *B. bassiana* conidia application and *N. californicus* release, under both laboratory and greenhouse conditions, substantially reduced the negative effects of the fungus on the predatory mite. These findings indicate that the combined use of these biological control agents requires a well-defined sequential application strategy to maximize *T. urticae* suppression by the predatory mite.

## Introduction

The combined use of biological control agents has been considered a strategy for effectively managing various agricultural pests, as multiple natural enemies coexist on shared hosts (Mills 2006). This strategy and its implications for biological control have been extensively studied in associations between invertebrate-pathogenic fungi and entomophagous insects across several crops and conditions (Roy and Pell 2000; Vázquez 2019; Quesada-Moraga et al. 2022). Several studies combining invertebrate fungal pathogens and predatory mites have shown promising results under laboratory conditions (Wu et al. 2014, 2016a) and also in the field (Vergel et al. 2011; Wu et al. 2018; Hernández-Valencia et al. 2024). Predatory mites can disperse fungal conidia within target pest populations, thereby increasing pathogen infection rates (Lin et al. 2019; Castillo-Ramírez et al. 2020). Conversely, the combined use of predatory mites and fungal pathogens may negatively affect predation, potentially leading to the displacement or exclusion of predatory mites (Chandler et al. 2005; Maniania et al. 2008; Wu et al. 2016b). Highly mite-pathogenic fungal strains can directly affect predatory mites through infection, reducing their longevity and fecundity or impairing predation efficacy due to behavioral changes (Duso et al. 2008; Seiedy et al. 2012; Ullah and Lim 2017). Additionally, high infection levels of phytophagous mites by the fungus may reduce prey availability, leading to declines in predatory mite populations through starvation or migration (Chandler et al. 2005).

Predatory mites of the family Phytoseiidae have been used as biological control agents to manage phytophagous mites across different regions of the world (McMurtry et al. 2015). Mites of the genus *Neoseiulus* have the two-spotted spider mite, *Tetranychus urticae* Koch (Tetranychidae), as a major target pray and are used in augmentative biological control programs in Latin American countries (Vásquez et al. 2023). Similarly, the fungus *Beauveria bassiana *(Balsamo) Vuillemin (Cordycipitaceae) has been commercialized in the region for short-term control of *T. urticae* through inundative releases (Li et al. 2010; Mascarin et al. 2019). In general, positive or negative interactions between *B. bassiana* and *Neoseiulus* species in combined applications are strongly related to the susceptibility of the predatory mite to infection by the selected fungal strain. Under laboratory conditions, highly virulent strains of *B. bassiana* targeting *T. urticae* have been reported to cause either no effect (Wu et al. 2016a) or significant negative effects (Michereff-Filho et al. 2022) on *Neoseiulus* adults exposed to fungal conidia. Additional factors, including fungal formulation, dosage, and the delivery strategy of both biocontrol agents under field conditions, may strongly influence the outcome of these interactions. A better understanding of these factors is therefore essential for the effective combined use of predatory mites and mite-pathogenic fungi.

We previously selected, under laboratory conditions, a highly virulent strain of *B. bassiana* against adults, immatures, and eggs of *T. urticae*, and demonstrated its detrimental effects on adults of three *Neoseiulus* species in arena bioassays. Our preliminary results further suggest that applying the fungus immediately before or after the release of predatory mites may reduce predator survival (Michereff-Filho et al. 2022). Similarly, Wu et al. (2016b) found no positive effect from the simultaneous use of the fungus *B. bassiana* and *Neoseiulus barkeri* (Hughes), to control *Frankliniella occidentalis* (Pergande) on greenhouse-grown cucumber plants, and the authors attributed these results to possible negative interactions between the two natural enemies. However, under field conditions, multiple factors may modulate predator-pathogen interactions, including prey and predator behavior, their distribution on the plants, pest density, plant architecture, and microenvironmental conditions. For example, predatory mites may avoid feeding on fungus-infected prey or seek shelter and alternative food sources in plant parts less exposed to fungal applications, thereby reducing their infection risk.

In this context, the primary objective of this study was to evaluate the effect of the combined use of *B. bassiana* and *Neoseiulus californicus* McGregor (Phytoseiidae) on the control of *T. urticae* populations on sweet pepper plants cultivated under greenhouse conditions. Additionally, we assessed a temporal selectivity strategy by releasing the predatory mite at different intervals after fungal application to optimize *T. urticae* control.

## Materials and methods

### Plants, mite colonies, and fungal preparation

*Tetranychus urticae* was reared on potted jack bean plants, *Canavalia ensiformis* (L.) DC. (Fabaceae), approximately 30–40 cm in height, under controlled laboratory conditions [26 ± 2 °C, 65 ± 5% relative humidity (RH), 12 h photo phase]. Plants were grown in 3 L pots containing a 2:1 mixture of autoclaved soil collected in field and commercial substrate (mix of *Pinus* bark, vermiculite, charcoal powder, and nutritional additives for vegetables) and kept in a greenhouse until use. Plants were replaced every 20 days, or as needed, to maintain the *T. urticae* colony. Jack bean plants were also used as hosts in laboratory bioassays.

For greenhouse experiments, sweet pepper plants, *Capsicum annuum* L. (Solanaceae) (cv. Dahra RX), were cultivated in 5 L pots containing a 3:1 mixture of soil and commercial substrate. Six days after transplanting, pest-free plants measuring 25–30 cm in height were used in the experiments.

The predatory mite *N. californicus* was obtained from a commercial supplier (Neomip Max^®^, Promip, Limeira, São Paulo, Brazil) and maintained on black plastic sheet arenas (15 × 10 cm) placed over water-saturated polyethylene foam (McMurtry and Scriven 1965; Souza-Pimentel et al. 2017). A moistened cotton barrier was placed around each arena to prevent mite escape. A jack bean leaf infested with *T. urticae* was added to each arena and replaced as needed. Dry pollen of the perennial wetland plant *Typha domingensis* Pers. (Typhaceae) was periodically supplied as an additional food source (Vacacela Ajila et al. 2019). The predatory mite colony was maintained under the same environmental conditions described above.

The fungal strain CG18 of *B. bassiana*, previously selected for high virulence against *T. urticae* (Michereff-Filho et al. 2022), is deposited in the Invertebrate-Associated Fungal Collection at Embrapa Genetic Resources and Biotechnology, Brasília, Federal District, Brazil. The strain was grown on potato dextrose agar (PDA) and maintained at 25 ± 0.5 °C with a 12 h photo phase. Aerial conidia used in laboratory and greenhouse experiments were produced on pre-cooked parboiled rice loaded into polypropylene bags, inoculated with a conidial suspension from the PDA cultures and incubated for 12 days under the same conditions. The fungus-colonized rice was pre-dried at room temperature for 48 h, and conidia were harvested by sieving. The harvested conidia were then dried over silica gel for 2 days at 24 ± 1 °C in hermetically sealed glass containers (3 L). Unformulated dry conidia had a concentration of approximately 1 × 10^11^ conidia/g and 6–6.5% moisture (equivalent to ca. 0.2 water activity). Conidial viability exceeded 95% at the time of use.

### Combined use of unformulated conidia of *B. bassiana* and *N. californicus* against *T. urticae* on sweet pepper plants under greenhouse conditions

The experiment was conducted in a glass-roofed greenhouse enclosed with anti-aphid mesh walls (8 m long x 4.5 m wide x 5 m high). Sixty-day-old sweet pepper plants (approximately 40 cm tall) were arranged on benches (0.5 m x 3.5 m). Each plant was infested with 20 *T. urticae* females collected from the laboratory colony two weeks before treatment application. Leaf segments of jack bean containing newly mated adult females were pinned to leaves in the middle region of the canopy of each sweet pepper plant. To prevent mite escape and intrusion of other predators, entomological glue (SprayTrap^®^, Promip, Limeira, São Paulo, Brazil) was applied to the inner upper edge of each pot.

For this greenhouse trial, 10 treatments were evaluated: (1) *T. urticae*-infested plants sprayed with unformulated *B. bassiana* conidia at two concentrations (1 × 10^8^ or 2 × 10^8^ conidia/mL in water + 0.05% v/v Tween 80); (2) delivery of 50 five-day-old *N. californicus* adults (35 females and 15 males) on plants without fungal application; and (3) the combination of *N. californicus* and *B. bassiana* at both fungal concentrations. Plants sprayed with water + surfactant (Tween 80) only served as the negative control. Fungal suspensions (50 mL per plant) were applied early in the morning using a manual pressure sprayer (Guarany^®^, 1.25 L capacity) equipped with a hollow-cone nozzle. Predators were collected from the laboratory stock with a soft-bristled brush and transferred to screw-cap microcentrifuge tubes. Each tube, containing the predators and a small leaf segment containing *T. urticae* eggs, was fixed between two branches in the middle third of the plant canopy, after which the lid was removed to allow predator dispersal. Tubes were monitored for 35 min after opened to ensure predators dispersal. In treatments combining the fungus and the predatory mite, *N. californicus* was released onto plants 6 h after fungal application. The experiment followed a randomized complete block design with five replications. Each bench represented one block (*n* = 5), with one plant per treatment per block, spaced 0.35 m apart.

*Tetranychus urticae* populations (adults, immatures, and eggs) were evaluated on each plant immediately before treatment application by visually inspecting three leaves. *Tetranychus urticae* and *N. californicus* individuals were subsequently counted at seven and 14 days after treatment application (Palevsky et al. 2008). For these evaluations, four leaves per plant were collected and all individuals were counted under a stereomicroscope (40x). During the experimental period, mean temperature and RH in the greenhouse were 28.1 ± 5.5 °C and 54.0 ± 6.0%, respectively.

### Sequential use of *B. bassiana* and *N. californicus* against *T. urticae*: temporal selectivity

Initially, we evaluated how the interval between fungal application and predator release affects the exposure of *N. californicus* to *B. bassiana* on *T. urticae*-infested plants under laboratory conditions. The experiment was conducted using potted jack bean plants with two fully expanded cotyledonary leaves, maintained in a climate-controlled room (25.1 ± 2.3 °C, 68.2 ± 1.4% RH, 12 h photoperiod). Each cotyledonary leaf was an experimental unit, which was infested with 30 *T. urticae* adult females one week before fungal application. Entomological glue was applied to the petiole of each leaf to control the movement of mites across leaves of the same plant. An aqueous suspension of *B. bassiana* conidia (1 × 10^8^ viable conidia/mL + 0.05% v/v Tween 80; 15 mL per plant) was applied using a manual pressure sprayer equipped with a hollow-cone nozzle. Predatory mites (25 adults per leaf) were released after the leaf surface had dried, either immediately or at two, four, and six days after spraying. Plants sprayed with water served as the negative control. To prevent mite escape, entomological glue was applied to the leaf petiole. Six days after predator release, the number of live and dead predators (adults and progeny) on each leaf was recorded under a stereomicroscope. The experiment followed a randomized block design with four replicates (one leaf per replicate). The six-day experimental period was chosen to allow the production of immature mites while preventing them from reaching reproductive maturity. The instantaneous growth rate (*ri*) was also calculated for each treatment to assess the potential impact of *B. bassiana* on *N. californicus* population dynamics.

**Fig. 1:**
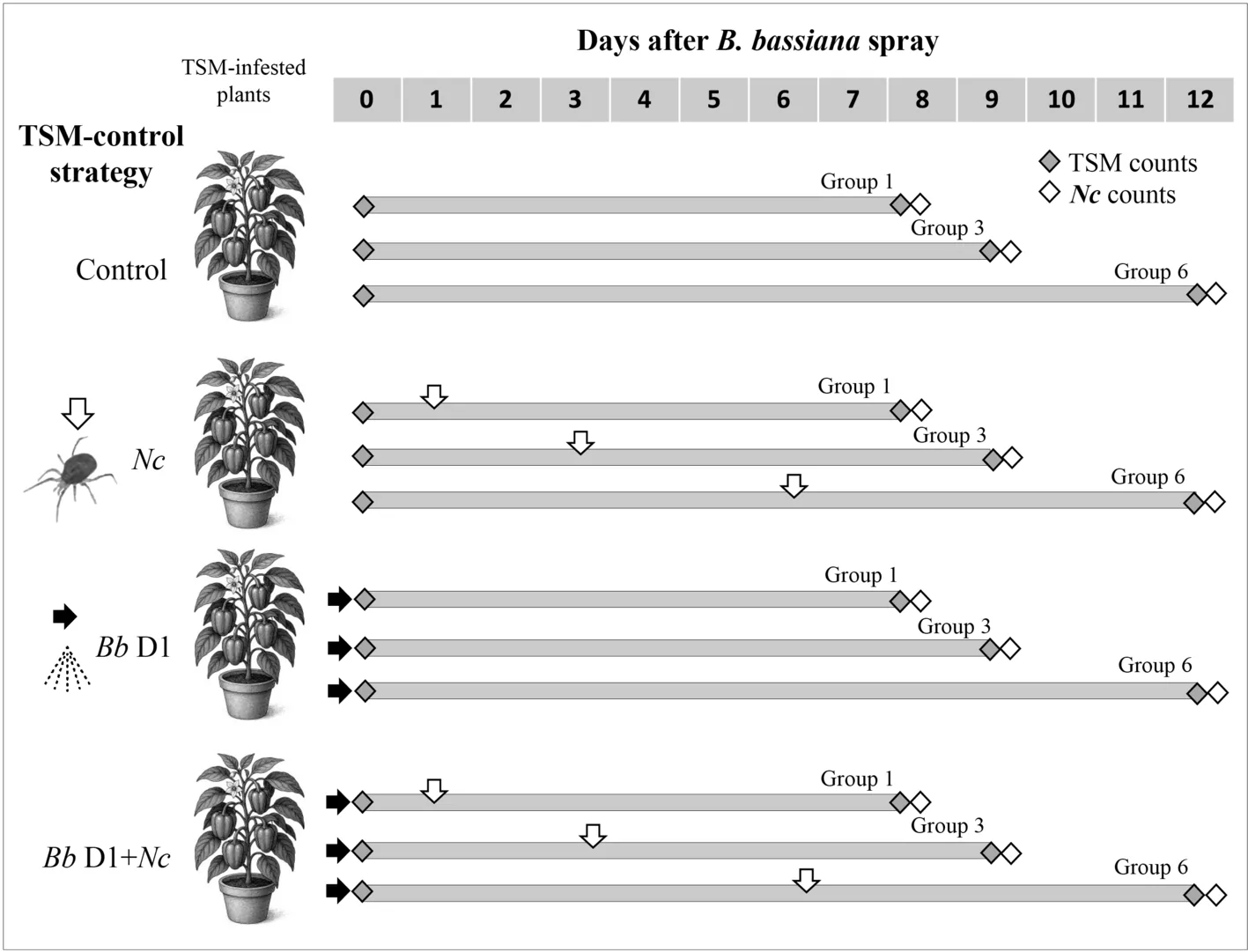
Schematic illustration of the experimental design for the sequential application of *Beauveria bassiana* CG18 (*Bb*; D1 = 1 × 10^8^ conidia/mL) and the release of *Neoseiulus californicus* (*Nc*) in a greenhouse. Black and white arrows indicate the application of fungal conidia and the release of the predatory mite (50 individuals per plant), respectively. Gray and white diamonds indicate the counts of adults, immatures, and eggs of *Tetranychus urticae* and the predatory mite

A second experiment was conducted in a greenhouse using three groups of 60-day-old sweet pepper plants grown individually in pots and arranged on benches. Each group (24 plants) was divided into four treatments of six plants (replicates) each, corresponding to three *T. urticae* control strategies (*N. californicus*, *B. bassiana*, and their combination) and an untreated control. The groups represented different intervals between fungal application and predator release [untreated (0), one, three, or six days after fungal application; Fig. 1]. All plants were infested with *T. urticae* by transferring 20 adult females per plant two weeks before treatment application. Entomological glue was applied to the inner upper edge of each pot to prevent mite escape. *Beauveria bassiana* was applied once at 1 × 10⁸ viable conidia/mL in water + 0.05% v/v Tween 80 (60 mL per plant), early in the morning, using a manual sprayer as previously described. Control plants were sprayed with distilled water + surfactant (Tween 80) only on the same day. Predatory mites (50 *N. californicus* individuals per plant) were released in the early morning following the same procedures described above.

*Tetranychus urticae* population density (adults, immatures, and eggs per leaf) was estimated one day before fungal application by inspecting three leaves per plant (from the upper, middle, and lower thirds) with a 10x pocket hand lens. Six days after predator release, adults, immatures, and eggs of both *T. urticae* and *N. californicus* were counted on all leaves of each plant under a stereomicroscope (40x) (Fig. 1). Mean temperature and RH during the experiment were 27.8 ± 4.2 °C and 57.0 ± 4.0%, respectively.

### Statistical analysis

The numbers of *T. urticae* and *N. californicus* adults, immatures, and eggs per leaf in greenhouse experiments involving the combined use of unformulated *B. bassiana* conidia and *N. californicus* were log-transformed [log(x + 1)] and subjected to analysis of variance (ANOVA) using a split-plot arrangement within a randomized block design, in which sub-plots corresponded to evaluation dates. Means were compared using Tukey′s HSD post hoc test (*p* < 0.05). Control efficacy (CE%) of *T. urticae* was estimated using the formula of Henderson and Tilton (1955).

In both experiments evaluating the sequential use of *B. bassiana* and *N. californicus*, treatments followed a factorial design with two factors: *T. urticae* control strategy and the interval between fungal application and predator release. Population density data for both mite species were log-transformed [log(x + 1)] and subjected to two-way ANOVA. For *T. urticae*, 12 treatment combinations were evaluated (control methods x predator release interval). For *N. californicus*, six treatment combinations were analyzed, excluding those without predator release. Treatment means were compared using Tukey′s HSD post hoc test (*p* < 0.05). When a significant interaction was detected, means were compared within each factor. CE% was also estimated based on changes in *T. urticae* populations between pre- and post-application evaluations.

The instantaneous growth rate (*ri*) for each treatment in the laboratory experiment combining fungus and predator was calculated according to the equation proposed by Walthall and Stark (1997), as follows:

*ri* = ln(Nf / N0) /Δt,

where Nf is the final number of live mites at the end of the experiment, N0 is the initial number of adult mites released, and Δt is the duration of the experiment. Positive *ri* values indicate population increase, *ri* = 0 denotes a stable population, and negative *ri* values indicate a declining population tending toward extinction when Nf = 0 (Stark and Banks 2003). The *ri* value was calculated for each treatment replicate. For statistical analysis, adult mortality (%), and *ri* were square-root transformed (√x + 1), whereas progeny per female of *N. californicus* were log-transformed [log(x + 1)]. Data were subjected to ANOVA, and treatment means were compared using Tukey′s HSD post hoc test (*p* < 0.05). All statistical analyses were performed using the SAS Statistical software (SAS Institute 2002).

## Results

### Combined use of unformulated conidia of *B. bassiana* and *N. californicus* against *T. urticae* on sweet pepper plants under greenhouse conditions

Populations of *T. urticae* adults (F_5,30_ = 1.13, *p* = 0.3744), immatures (F_3,28_ = 0.38, *p* = 0.8199), and eggs (F_3,28_= 0.65, *p* = 0.6652) were similar among plots after two weeks of infestation, before treatment application (Tables 1 and 2), indicating homogeneous colonization of plants by the mite. Significant interactions between *T. urticae* control strategy and evaluation date were detected for adults (F_5,20_ = 3.20, *p* = 0.0276), immatures (F_5,2 0_= 48.14, *p* < 0.0001), and eggs (F_5,2 0_= 22.68, *p* < 0.0001). A decline in *T. urticae* populations was observed seven days after the release of *N. californicus*, application of *B. bassiana*, and their combination at both conidial concentrations. After 14 days, CE of the predatory mite alone decreased, and *T. urticae* populations did not differ from those in untreated plants (Tables 1 and 2).

**Table 1:** Mean (± standard error) number of *Tetranychus urticae* adults and immatures per leaf on sweet pepper plants treated with unformulated conidia of *Beauveria bassiana* and the predatory mite *Neoseiulus californicus* in a greenhouse

Treatments^a^	Days after treatment^b^									
	*Tetranychus urticae* adults			CE (%)^c^		*Tetranychus urticae* immatures			CE (%)^c^	
	Before	7	14	7	14	Before	7	14	7	14
Control	5.6 ± 0.93 a	8.6 ± 0.83 aB	19.8 ± 1.20 aA	-	-	13.1 ± 2.16 a	20.1 ± 1.93 aB	46.2 ± 2.80 aA	-	-
*Nc*	6.9 ± 0.44 a	4.9 ± 0.78 bB	17.8 ± 0.98 aA	53.20	27.08	16.1 ± 1.03 a	11.5 ± 1.81 bB	41.5 ± 2.29 aA	53.61	27.08
*Bb* D1	6.6 ± 0.52 a	4.1 ± 0.87 bB	11.0 ± 1.28 bA	59.30	52.84	15.4 ± 1.21 a	9.6 ± 2.02 bB	25.7 ± 2.99 bA	59.23	52.88
*Bb* D2	5.7 ± 0.74 a	1.7 ± 0.48 cB	4.8 ± 1.05 cA	81.06	76.15	13.3 ± 1.73 a	4.5 ± 1.11 cB	11.2 ± 3.69 cA	78.00	76.16
*Bb* D1 + *Nc*	6.0 ± 0.61 a	3.5 ± 1.06 bcB	8.8 ± 1.33 bA	62.06	58.35	14.0 ± 1.43 a	2.7 ± 2.09 cB	10.5 ± 2.80 cA	87.95	79.89
*Bb* D2 + *Nc*	6.3 ± 0.59 a	1.6 ± 0.89 cB	4.5 ± 1.11 cA	81.97	79.85	14.7 ± 1.37 a	5.8 ± 1.54 bcB	15.9 ± 3.50 cA	74.31	67.78

^a^ *Nc – Neoseiulus californicus* (50 adults per plant); *Bb* – *Beauveria bassiana* CG18 applied at 60 mL per plant at concentrations of 1 × 10^8^ (D1) and 2 × 10^8^ (D2) conidia/mL

^b^ Means followed by different lowercase letters between treatments (columns) or uppercase letters between evaluation days (rows) differ significantly according to Tukey’s HSD post hoc test (*p* < 0.05)

^c^ CE (%): Control efficacy calculated according to Henderson and Tilton (1955)

**Table 2:** Mean (± standard error) number of *Tetranychus urticae* eggs per leaf on sweet pepper plants treated with unformulated conidia of *Beauveria bassiana* and the predatory mite *Neoseiulus californicus* in a greenhouse

Treatments^a^	Days after treatment^b^			CE (%)^c^	
	Before	7	14	7	14
Control	8.5 ± 2.09 a	16.9 ± 3.75 aB	38.9 ± 3.27 aA	-	-
*Nc*	8.7 ± 1.47 a	6.7 ± 1.07 bB	32.9 ± 2.15 aA	61.49	17.62
*Bb* D1	10.5 ± 1.61 a	7.5 ± 1.30 bB	24.9 ± 1.43 bA	64.34	48.40
*Bb* D2	7.9 ± 2.47 a	3.6 ± 1.15 cB	17.0 ± 1.26 cA	77.20	53.26
*Bb* D1 + *Nc*	9.0 ± 2.09 a	2.8 ± 1.40 cB	14.7 ± 2.07 cA	84.44	64.39
*Bb* D2 + *Nc*	8.1 ± 1.95 a	4.3 ± 1.36 bcB	18.1 ± 1.32 cA	73.26	51.41

^a^ *Nc – Neoseiulus californicus* (50 adults per plant), *Bb* – *Beauveria bassiana* CG18 applied at 60 mL per plant at concentrations of 1 × 10^8^ (D1) and 2 × 10^8^ (D2) conidia/mL

^b^ Means followed by different lowercase letters between treatments (columns) or uppercase letters between evaluation days (rows) differ significantly according to Tukey’s HSD post hoc test (*p* < 0.05)

^c^ Control efficacy calculated according to Henderson and Tilton (1955)

**Fig. 2:**
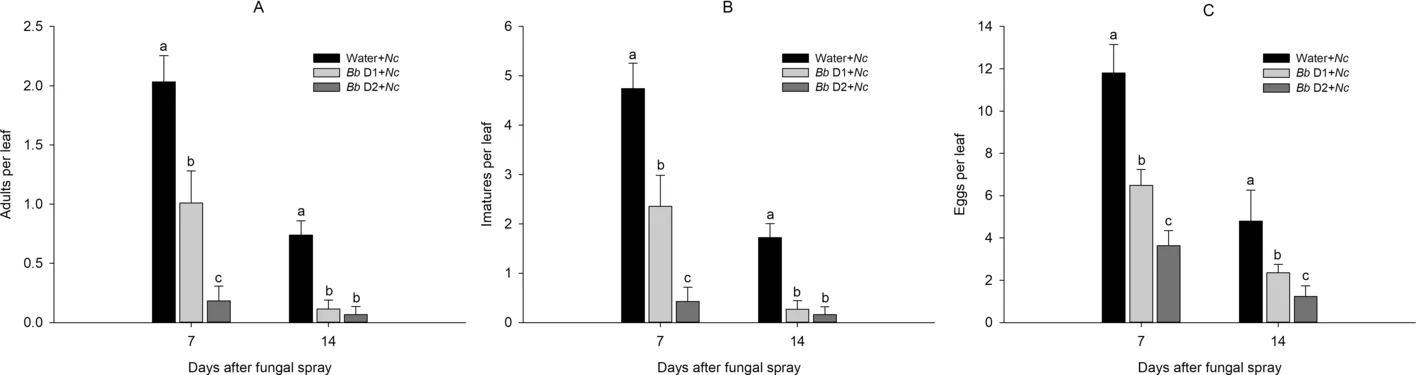
Mean (± standard) number of adults (**A**), imatures (**B**), and eggs (**C**) of the predatory mite *Neoseiulus californicus* per leaf on sweet pepper plants treated with unformulated conidia of *Beauveria bassiana* in a greenhouse at 7 and 14 days after fungal application. *Nc* – *Neoseiulus californicus* (50 adults per plant); *Bb* – *Beauveria bassiana* CG18 applied at 50 mL per plant at concentrations of 1 × 10^8^ (D1) and 2 × 10^8^ (D2) conidia/mL. Means followed by different letters within each evaluation date differ significantly according to Tukey’s HSD post hoc test (*p* < 0.05). Data were log-transformed [log(x + 1)] before statistical analyses

The populations of *N. californicus* in plants not treated with *B. bassiana* reached 2.0 ± 0.2 adults, 4.7 ± 0.5 immatures, and 11.8 ± 1.4 eggs per leaf after seven days (Fig. 2). A decline in all developmental stages of *N. californicus* was observed after 14 days in plants where the predatory mite was released. The numbers of adults, immatures, and eggs were significantly lower in plants sprayed with *B. bassiana* than in unsprayed plants (adults: F_2,8_ = 70.45, *p* = 0.0148; immatures: F_2,88_ = 70.45, *p* < 0.0001; eggs: F_2,8_ = 68.83, *p* < 0.0001). The interaction between fungal dose and evaluation date was significant for adults (F_2,8_ = 7.35, *p* = 0.0154) and immatures (F_2,8_ = 8.93, *p* = 0.0092).

Seven days after fungal application, the number of *N. californicus* adults and immatures was lower in plants treated with the higher conidial dose than in those treated with the lower dose. This difference was not detected after 14 days. The higher conidial dose of *B. bassiana* also reduced egg numbers of *N. californicus* on the leaves (F_2,8_ = 68.83, *p* < 0.0001), and this effect persisted until the end of the experimental period. At the higher fungal dose, predatory mite population decreased by more than 90% for adults and immatures and 74.2% for eggs relative to unsprayed plants after 14 days.

### Sequential use of *B. bassiana* and *N. californicus* against *T. urticae*: temporal selectivity

In the first laboratory experiment, significant differences in predatory mite mortality were observed among the intervals between fungal application and mite release (F_4,12_ = 5.67, *p* = 0.0084). Mortality of *N. californicus* adults exceeded > 79% when the predatory mite was released immediately after fungal application, decreasing to an average of 25% when conidia were applied six days before mite release (Table 3). The number of immatures (F_4,12_ = 8.91, *p* = 0.0014), eggs (F_4,12_ = 13.55, *p* = 0.0002) and *ri* (F_4,12_ = 10.56, *p* = 0.0007) were also affected by the different intervals between *B. bassiana* application and mite release. Egg and immature counts were lower at shorter exposure intervals to fungal conidia. No differences in eggs or immature numbers per female, or in *ri*, were observed when the interval between fungal application and mite release exceeded four days (Table 3).

**Table 3:** Mean (± standard error) adult mortality (%), number of immatures and eggs per female, and instantaneous growth rate (*ri*) of the predatory mite *Neoseiulus californicus* exposed to *Beauveria bassiana* conidia on *Canavalia ensiformis* at different intervals between fungal application and predator release, evaluated six days after release under laboratory conditions

Mite release interval	Adult mortality (%)^a^	Immatures / female^a^	Eggs / female^a^	*ri* ^a^
Control^b^	7.0 ± 5.74 b	3.6 ± 0.45 a	1.4 ± 0.14 a	0.35 ± 0.02 a
Immediately after spraying	79.0 ± 5.26 a	1.1 ± 0.36 b	0.7 ± 0.26 b	0.12 ± 0.05 b
2 days after spraying	47.0 ± 10.76 ab	1.3 ± 0.13 b	0.7 ± 0.13 b	0.18 ± 0.02 b
4 days after spraying	38.0 ± 12.91 ab	0.8 ± 0.60 b	1.3 ± 0.29 a	0.18 ± 0.05 b
6 days after spraying	25.0 ± 8.66 ab	2.5 ± 0.37 a	1.5 ± 0.29 a	0.29 ± 0.01 a

^a^ Means followed by different letters between treatments (columns) differ significantly according to Tukey’s HSD post hoc test (*p* < 0.05). Data were square-root transformed (√(x + 1)) before statistical analyses

^b^ Plants sprayed only with water + 0.05% v/v Tween 80

In the greenhouse experiment, the initial *T. urticae* infestation did not differ among sweet pepper plants across treatments (adults: F_11,55_ = 0.48, *p* = 0.9085; immatures: F_11,55_ = 1.17, *p* = 0.3300; eggs: F_11,55_ = 0.35, *p* = 0.9702), confirming homogeneous pest distribution. After treatment, infestation levels differed significantly among treatments for adults (F_3,30_ = 68.44, *p* < 0.0001), immatures (F_3,30_ = 93.05, *p* < 0.0001), and eggs (F_3,30_ = 105.27, *p* < 0.0001). Significant effects of the interval between fungal application and predator release were also observed for adults (F₂,₃₀ = 19.37, *p* = 0.0004), immatures (F_2,30_ = 76.85, *p* < 0.0001), and eggs (F_2,30_ = 62.95, *p* < 0.0001). Application of *B. bassiana* significantly reduced *T. urticae* adult populations, whereas plants treated with *N. californicus* alone did not differ from untreated plants (Table 4).

**Table 4:** Mean (± standard error) number of *Tetranychus urticae* adults per leaf on sweet pepper plants treated with unformulated conidia of *Beauveria bassiana* and the predatory mite *Neoseiulus californicus*, at different intervals between fungal application and predator release in a greenhouse

Treatments^a^	*T. urticae* adults^b^								
	*Nc* release intervals (days)^c^						CE (%)^d^		
	Initial infestation	1 day	Initial infestation	3 days	Initial infestation	6 days	1 day interval	3 days interval	6 days interval
Control	5.60 ± 0.16 a	12.20 ± 3.03 aC	5.44 ± 0.34 a	27.42 ± 1.54 aB	5.63 ± 0.17 a	52.70 ± 6.05 aA	-	-	-
*Nc*	5.39 ± 0.10 a	9.30 ± 0.61 aC	5.51 ± 0.10 a	23.21 ± 0.91 aB	6.20 ± 2.80 a	53.24 ± 2.36 aA	20.80	16.43	8.26
*Bb* D1	5.57 ± 0.55 a	5.23 ± 0.45 bC	6.03 ± 0.59 a	15.45 ± 0.55 bB	5.42 ± 0.72 a	24.31 ± 1.17 bA	56.90	49.17	40.86
*Bb* D1 + *Nc*	6.11 ± 0.31 a	4.81 ± 0.15 bC	5.75 ± 0.48 a	14.37 ± 0.30 bB	5.78 ± 0.30 a	24.52 ± 1.03 bA	63.83	50.42	44.07

^a^ *Nc – Neoseiulus californicus* (50 adults per plant), *Bb* – *Beauveria bassiana* CG18 applied at 50 mL per plant at a concentration of 1 × 10^8^ conidia/mL (D1)

^b^ Means followed by different lowercase letters between treatments (columns) or uppercase letters between intervals (rows) differ significantly according to Tukey’s HSD post hoc test (*p* < 0.05). Data were log-transformed [log(x + 1)] before statistical analyses

^c^ *Nc* release intervals of 1, 3, and 6 days after *Bb* application (evaluations performed at 7, 9, and 12 days after initial infestation, respectively)

^d^ Control efficacy calculated according to Henderson and Tilton (1955)

A significant interaction between the application interval of the biological control agents and the *T. urticae* control strategy was observed for immatures (F_6,30_ = 12.15, *p* < 0.0001) and eggs (F_6,30_ = 13.72, *p* < 0.0001), indicating that treatment effects on pest reduction varied across developmental stages. Plants treated with *N. californicus* alone, or with *B. bassiana* alone or in combination, showed marked reductions in *T. urticae* egg and immature densities relative to the control. The combined application of *B. bassiana* and *N. californicus* resulted in lower egg and immature infestations than the use of either natural enemy alone when the predator was released six days after fungal application (Tables 5 and 6).

**Table 5:** Mean (± standard error) number of *Tetranychus urticae* immatures per leaf on sweet pepper plants treated with unformulated conidia of *Beauveria bassiana* and the predatory mite *Neoseiulus californicus*, at different intervals between fungal application and predator release in a greenhouse

Treatments^a^	*T. urticae* immatures^b^								
	*Nc* release intervals (days)^c^						CE (%)^d^		
	Initial infestation	1 day	Initial infestation	3 days	Initial infestation	6 days	1 day interval	3 days interval	6 days interval
Control	2.92 ± 0.22 a	9.88 ± 0.81 aC	3.43 ± 0.51 a	24.70 ± 1.07 aB	3.65 ± 0.59 a	39.52 ± 1.43 aA	-	-	-
*Nc*	2.95 ± 0.24 a	4.43 ± 0.34 bC	3.11 ± 0.33 a	12.30 ± 0.88 bB	2.86 ± 0.37 a	19.33 ± 0.75 bA	55.62	45.08	37.58
*Bb* D1	3.35 ± 0.44 a	5.34 ± 0.41 bC	3.19 ± 0.29 a	11.61 ± 0.71 bB	3.48 ± 0.73 a	22.82 ± 0.96 bA	52.89	49.46	39.44
*Bb* D1 + *Nc*	3.54 ± 0.39 a	5.62 ± 0.36 bB	2.79 ± 0.46 a	10.03 ± 0.59 bA	3.07 ± 0.42 a	13.43 ± 0.37 cA	53.08	50.08	59.60

^a^ *Nc – Neoseiulus californicus* (50 adults per plant), *Bb* – *Beauveria bassiana* CG18 applied at 50 mL per plant at a concentration of 1 × 10^8^ conidia/mL (D1)

^b^ Means followed by different lowercase letters between treatments (columns) or uppercase letters between intervals (rows) differ significantly according to Tukey’s HSD post hoc test (*p* < 0.05). Data were log-transformed [log(x + 1)] before statistical analyses

^c^ *Nc* release intervals of 1, 3 and 6 days after *Bb* application (evaluations at 7, 9 and 12 days after initial infestation, respectively)

^d^ Control efficacy calculated according to Henderson and Tilton (1955)

**Table 6:** Mean (± standard error) number of *Tetranychus urticae* eggs per leaf on sweet pepper plants treated with unformulated conidia of *Beauveria bassiana* and the predatory mite *Neoseiulus californicus*, at different intervals between fungal application and predator release in a greenhouse

Treatments^a^	*T. urticae* eggs^b^								
	*Nc* release intervals (days)^c^						CE (%)^d^		
	Initial infestation	1 day	Initial infestation	3 days	Initial infestation	6 days	1 day interval	3 days interval	6 days interval
Control	7.47 ± 0.18 a	20.91 ± 1.27 aC	8.23 ± 0.40 a	34.87 ± 1.91 aB	7.68 ± 0.44 a	48.83 ± 2.55 aA	-	-	-
*Nc*	7.58 ± 0.24 a	7.22 ± 0.67 bC	7.80 ± 0.26 a	13.02 ± 0.99 bB	7.33 ± 0.80 a	29.65 ± 0.32 bA	65.97	60.60	36.25
*Bb* D1	8.42 ± 0.29 a	9.83 ± 0.82 bC	7.57 ± 0.20 a	13.89 ± 0.49 bB	8.23 ± 0.55 a	29.54 ± 0.51 bA	58.29	56.69	44.01
*Bb* D1 + *Nc*	7.60 ± 0.40 a	8.55 ± 0.59 bB	7.80 ± 0.25 a	13.32 ± 0.79 bA	7.45 ± 0.36 a	15.94 ± 0.86 cA	59.81	59.70	66.36

^a^ *Nc – Neoseiulus californicus* (50 adults per plant); *Bb* – *Beauveria bassiana* CG18 applied at 50 mL per plant at a concentration of 1 × 10^8^ conidia/mL (D1)

^b^ Means followed by different lowercase letters between treatments (columns) or uppercase letters between intervals (rows) differ significantly according to Tukey’s HSD post hoc test (*p* < 0.05). Data were log-transformed [log(x + 1)] before statistical analyses

^c^ *Nc* release intervals of 1, 3 and 6 days after *Bb* application (evaluations at 7, 9 and 12 days after initial infestation, respectively)

^d^ Control efficacy calculated according to Henderson and Tilton (1955)

Populations of *N. californicus* on plants treated with *B. bassiana* varied according to the interval between fungal application and predator release for adults (F_2,10_ = 39.79, *p* < 0.0001), immatures (F_2,10_ = 15.81, *p* = 0.0008), and eggs (F_2,10_ = 82.73, *p* < 0.0001). Adult, immature, and egg counts of *N. californicus* were significantly lower when predators were released one or three days after fungal application than in untreated plants. Increasing the interval between *B. bassiana* application and predator release improved predatory mite establishment on sweet pepper plants (Table 7).

**Table 7:** Mean (± standard error) number of adults, immatures, and eggs per leaf and estimated population decrease of the predatory mite *Neoseiulus californicus* exposed to *Beauveria bassiana* conidia on sweet pepper plants at different intervals between fungal application and predator release

Mite release interval	Treatments^a^		Mite population decrease (%)^c^
	*Nc*	*Bb* D1 + Nc	
***Nc*** **adults per leaf**^b^			
1 day after spraying	1.58 ± 0.30 aA	0.40 ± 0.07 bB	75.0
3 days after spraying	2.37 ± 0.45 aA	1.07 ± 0.20 bA	55.0
6 days after spraying	3.17 ± 0.60 aA	2.22 ± 0.42 aA	30.0
***Nc*** **immatures per leaf**^b^			
1 day after spraying	1.24 ± 0.35 aA	0.25 ± 0.07 bC	80.0
3 days after spraying	1.75 ± 0.50 aA	0.88 ± 0.25 bB	50.0
6 days after spraying	2.33 ± 0.67 aA	1.98 ± 0.57 aA	15.0
***Nc*** **eggs per leaf**^**2**^			
1 day after spraying	3.65 ± 0.31 aC	0.81 ± 0.07 bC	78.0
3 days after spraying	5.37 ± 0.45 aB	2.69 ± 0.22 bB	50.0
6 days after spraying	7.17 ± 0.60 aA	6.45 ± 0.54 aA	10.0

^a^ *Nc – Neoseiulus californicus* (50 adults per plant); *Bb* – *Beauveria bassiana* CG18 applied at 50 mL per plant at a concentration of 1 × 10^8^ conidia/mL (D1)

^b^ Means followed by different lowercase letters between treatments (rows) or uppercase letters between intervals (columns) differ significantly according to Tukey’s HSD post hoc test (*p* < 0.05). Data were log-transformed [log(x + 1)] before statistical analyses

^c^ Estimated reduction in *Nc* population based on adult density on plants treated only with the predator

## Discussion

The polyphagous behavior and high reproductive potential of *T. urticae*, combined with optimal conditions found in greenhouses, promote rapid population growth and severe damage to several horticultural crops (Praslička and Huszar 2004; Vacante 2015; Rioja et al. 2017). Successful use of biological control agents against *T. urticae* in heavily infested greenhouse crops, such as predatory mites or mite-pathogenic fungi, typically requires high release rates and repeated applications to suppress population outbreaks (Blumel and Walzer 2002; Gatarayiha et al. 2011; Ullah and Lim 2015; Howell and Daugovish 2016; Lemaire et al. 2025). The ability of some invertebrate-pathogenic fungi to infect multiple hosts increases the risk of non-target effects on predator survival when these agents are used in combination. We previously showed that the combined use of *B. bassiana* and predatory mites of the genus *Neoseiulus* had detrimental effects on predator survival and progeny under laboratory conditions (Michereff-Filho et al. 2022). In the present study, the interval of six days between the application of *B. bassiana* conidia and the release of *N. californicus*, under both laboratory and greenhouse conditions, drastically reduced these negative effects on the predatory mite. These findings suggest that the combined use of these biological control agents should follow a well-defined sequential application strategy.

Promising results from the use of *N. californicus* against *T. urticae* on pepper plants have been reported in protected cultivation systems (Palevsky et al. 2008; Weintraub and Palevsky 2008). In general, inundative releases at predator-prey ratios ranging from 1:3 to 1:10 are considered crucial for achieving high levels of *T. urticae* control (Greco et al. 2005; Fraulo and Liburd 2007; Weintraub and Palevsky 2008). In the present study, *N. californicus* partially reduced *T. urticae* populations on heavily infested sweet pepper plants under greenhouse conditions, with significant effects on different developmental stages after one week, and greater efficacy against eggs than against adults and immatures. The lower impact on *T. urticae* adults was expected, given the predator’s preference for immatures and eggs of tetranychids (Blackwood et al. 2001; Canlas et al. 2006; Akyazi et al. 2019) and its reduced ability to capture adult individuals (Rahmani et al. 2016). The short-term suppression of *T. urticae* can be attributed to the sharp decline in predator numbers, followed by rapid resurgence of the pest population two weeks after predator release.

The lack of persistence of *N. californicus* on plants remains unclear, but may be associated with adverse greenhouse conditions, such as elevated temperatures (> 35 °C), low RH (< 45%) (Barber et al. 2003; Gotoh et al. 2004; Weintraub and Palevsky 2008), and early plant developmental stage (low number of leaves), which may have created unfavorable conditions for predator establishment and survival. These results indicate that, when used alone to control high *T. urticae* infestations, *N. californicus* requires more frequent releases throughout the crop cycle, consistent with observations by Howell and Daugovish (2016) in strawberry production.

Despite unfavorable environmental conditions in the greenhouse, the CG18 strain of *B. bassiana* was effective against the two-spotted spider mite. Higher conidial doses resulted in improved control across all developmental stages of *T. urticae*. Similar population reductions have been reported for other *B. bassiana* isolates targeting this pest (Chandler et al. 2005; Shi et al. 2008; Gatarayiha et al. 2011; Ullah and Lim 2017), highlighting the potential of the CG18 strain for integrated management of *T. urticae* in greenhouse horticultural systems. Although the predator alone reduced *T. urticae* adult and immature numbers during the first week after release, the greater efficacy observed in the combined treatment was primarily driven by the fungal pathogen. Notably, the abundance of all developmental stages of the predatory mite declined sharply in treatments where the fungus was applied simultaneously, suggesting a direct (death by infection) or indirect (prey availability or prey repellency) negative effect on the predator. It is important to note that high conidial doses are required to suppress *T. urticae* populations on heavily infested plants, which intensifies the negative effect on predatory mite populations.

Strategies to mitigate the detrimental effects of the fungus on the predatory mite include selecting fungal strains with greater selectivity toward the predator (Wu et al. 2014, 2016a, 2018), as well as strategies that reduce pathogen-natural enemy contact, such as temporal selectivity (Wu et al. 2017; Hernández-Valencia et al. 2024). In the present study, laboratory results obtained on jack bean plants showed that *B. bassiana* had a pronounced negative effect on *N. californicus*, reducing adult survival, immature and egg production per female, and *ri* when predators were released immediately after fungal application, consistent with arena bioassay observations (Michereff-Filho et al. 2022). This negative effect decreased as the interval between the fungal application and predator release increased.

A similar pattern was observed in the greenhouse experiment with sweet pepper plants. At the six-day interval, adult, immature, and egg counts of *N. californicus* per leaf did not differ between treatments with and without the fungus. The smaller reduction in *N. californicus* populations at this interval resulted in greater *T. urticae* suppression, particularly of immatures and eggs. When either agent was used alone, *T. urticae* populations increased as the application interval lengthened; however, in the combined treatment, populations remained stable at the six-day interval. Relative to the initial infestation, *T. urticae* counts increased 4.1- and 3.6-fold when the predator and *B. bassiana* were used alone, respectively, compared with 2.1-fold in the combined treatment. For immatures, the populations increased 6.7-, 5.9-, and 4.4-fold, when the predatory mite, the fungus, and their combination were used, respectively. This difference was not observed for adults, reflecting the lower predation capacity of *N. californicus* on this stage (Rahmani et al. 2016).

Our results demonstrated that the combined use of *B. bassiana* and the predatory mite *N. californicus* in a single application reduced the population growth of *T. urticae* compared with untreated plants, but did not provide effective long-term suppression of the pest. Nevertheless, several measures could improve the efficacy of these natural enemies against the two-spotted spider mite during hot and dry seasons. For instance, reducing heat stress in greenhouses by installing shading nets and using a fogging system combined with forced ventilation may decrease temperature, increase relative humidity, and create more favorable conditions for both biological control agents. In addition, delivering the biocontrol agents at lower pest densities (less than 10 mites per leaf), using high doses of fungal conidia, keeping an appropriate interval (6–7 days) between fungal application and predator release, and performing repeated applications could constitute an effective integrated strategy. Such an approach would minimize negative interactions between the biological control agents while maximizing their complementary effects on *T. urticae* suppression. Further studies are needed to optimize and validate this sequential application strategy for the integrated biological control of the two-spotted spider mite in greenhouse.

## Conclusion

High doses of *B. bassiana* conidia provided a reduction on *T. urticae* populations growth on infested sweet pepper plants under greenhouse conditions; however, the fungus was detrimental to the predatory mite *N. californicus* when both agents were applied in combination. In contrast, sequential applications of the biological control agents, with a minimum interval between applications, reduced the negative effects of the fungus on the predator and resulted in more sustained suppression of *T. urticae* populations. Application of the CG18 strain of *B. bassiana* as an aqueous conidial suspension at 1 × 10⁸ conidia/mL, followed by the release of *N. californicus* six days later, proved effective for *T. urticae* control. These findings support the development of improved strategies for integrating these two important natural enemies of tetranychids in protected cropping systems.

## Data Availability

The datasets generated during and/or analysed during the current study are available from the corresponding author on reasonable request.

## References

[CR1] Akyazi R, Soysal M, Altunç YE (2019) The prey-stage preferences of *Amblyseius swirskii* Athias-Henriot and *Neoseiulus californicus* (McGregor) (Mesostigmata: Phytoseiidae), between egg and nymph stages of *Tetranychus urticae* Koch (Trombidiformes: Tetranychidae). Plant Prot Bull 59(1):37–42. 10.16955/bitkorb.438910

[CR2] Barber A, Campbell CAM, Crane H, Lilley R, Tregidga E (2003) Biocontrol of two-spotted spider mite *Tetranychus urticae* on dwarf hops by the phytoseiid mites *Phytoseiulus persimilis* and *Neoseiulus californicus*. Biocontrol Sci Technol 13(3):275–284. 10.1080/0958315031000110300

[CR3] Blackwood JS, Schausberger P, Croft BA (2001) Prey-stage preference in generalist and specialist phytoseiid mites (Acari: Phytoseiidae) when offered *Tetranychus urticae* (Acari: Tetranychidae) eggs and larvae. Environ Entomol 30(6):1103–1111. 10.1603/0046-225X-30.6.1103

[CR4] Blumel S, Walzer A (2002) Efficacy of different release strategies of *Neoseiulus californicus* McGregor and *Phytoseiulus persimilis* Athias Henriot (Acari: Phytoseiidae) for the control of two-spotted spider mite (*Tetranychus urticae* Koch) on greenhouse cut roses. Syst Appl Acarol 7:35–48. 10.11158/saa.7.1.5

[CR5] Canlas LZ, Amano H, Ochiai N, Takeda M (2006) Biology and the predation Japannese strain of *Neoseiulus californicus* McGregor (Acari: Phytoseiidae). Syst Appl Acarol 11(2):141–157. 10.11158/saa.11.2.2

[CR6] Castillo-Ramírez O, Guzmán-Franco AW, Santillán-Galicia MT, Tamayo-Mejía F (2020) Interaction between predatory mites (Acari: Phytoseiidae) and entomopathogenic fungi in *Tetranychus urticae* populations. Biocontrol 65:433–445. 10.1007/s10526-020-10004-3

[CR7] Chandler D, Davidson G, Jakobson RJ (2005) Laboratory and glasshouse evaluation of entomopathogenic fungi against the two-spotted spider mite, *Tetranychus urticae* (Acari: Tetranychidae), on tomato, *Lycopersicon esculentum*. Biocontrol Sci Technol 15(1):37–54. 10.1080/09583150410001720617

[CR8] Duso C, Malagnini V, Pozzebon A, Castagnoli M, Liguori M, Simoni S (2008) Comparative toxicity of botanical and reduced-risk insecticides to Mediterranean populations of *Tetranychus urticae* and *Phytoseiulus persimilis* (Acari: Tetranychidae, Phytoseiidae). Biol Control 47:16–21. 10.1016/j.biocontrol.2008.06.011

[CR9] Fraulo AB, Liburd OE (2007) Biological control of two-spotted spider mite, *Tetranychus urticae*, with predatory mite, *Neoseiulus californicus*, in strawberries. Exp Appl Acarol 43:109–119. 10.1007/s10493-007-9109-7 17924197

[CR10] Gatarayiha MC, Laing MD, Miller RM (2011) Field evaluation of *Beauveria bassiana* efficacy for the control of *Tetranychus urticae* Koch (Acari: Tetranychidae). J Appl Entomol 135:582–592. 10.1111/j.1439-0418.2010.01569.x

[CR11] Gotoh T, Yamaguchi K, Mori K (2004) Effect of temperature on life history of the predatory mite *Amblyseius* (*Neoseiulus*) *californicus* (Acari: Phytoseiidae). Exp Appl Acarol 32:15–30. 10.1023/b:Appa.0000018192.91930.49 15139269

[CR12] Greco NM, Sanchez NE, Liljesthrom GG (2005) *Neoseiulus californicus* (Acari: Phytoseiidae) as a potential control agent of *Tetranychus urticae* (Acari: Tetranychidae): effect of pest/predator ratio on pest abundance on strawberry. Exp Appl Acarol 37:57–66. 10.1007/s10493-005-0067-7 16180072

[CR13] Henderson CF, Tilton EW (1955) Tests with acaricides against the brown wheat mite. J Econ Entomol 48(2):157–161. 10.1093/jee/48.2.157

[CR14] Hernández-Valencia V, Santillán-Galicia MT, Guzmán-Franco AW, Rodríguez-Leyva E, Santillán-Ortega C (2024) Combined application of entomopathogenic fungi and predatory mites for biological control of *Tetranychus urticae* on chrysanthemum. Pest Manag Sci 80(9):4199–4206. 10.1002/ps.8123 38597427

[CR15] Howell AD, Daugovish O (2016) Biocontrol of spider mites in California strawberry production. Int J Fruit Sci 16(1):169–177. 10.1080/15538362.2016.1195316

[CR16] Lemaire E, Mccune F, Roy M, Bourgeois G, Fournier V (2025) Efficacy of releases of phytoseiid mites (Acari: Phytoseiidae) on spider mite control on raspberry in high tunnel production, and implication on control strategy through a population model of *Tetranychus mcdanieli* (Acari: Tetranychidae). Biol Control 206:105799. 10.1016/j.biocontrol.2025.105799

[CR17] Li Z, Alves SB, Roberts DW, Fan M, Delalibera I Jr., Tang J, Lopes RB, Faria M, Rangel DEN (2010) Biological control of insects in Brazil and China: history, current programs and reasons for their successes using entomopathogenic fungi. Biocontrol Sci Technol 20(2):117–136. 10.1080/09583150903431665

[CR18] Lin G, Guertin C, Di Paolo SA, Todorova S, Brodeur J (2019) Phytoseiid predatory mites can disperse entomopathogenic fungi to prey patches. Sci Rep 9(1):19435. 10.1038/s41598-019-55499-8 PMC6923365 31857623

[CR19] Maniania NK, Bugeme DM, Wekesa VW, Delalibera I Jr., Knapp M (2008) Role of entomopathogenic fungi in the control of *Tetranychus evansi* and *Tetranychus urticae* (Acari: Tetranychidae), pests of horticultural crops. Exp Appl Acarol 46:259–274. 10.1007/s10493-008-9180-8 18685956

[CR20] Mascarin GM, Lopes RB, Delalibera I Jr, Fernandes EKK, Luz C, Faria M (2019) Current status and perspectives of fungal entomopathogens used for microbial control of arthropod pests in Brazil. J Invert Pathol 165:46–53. 10.1016/j.jip.2018.01.001 29339191

[CR21] McMurtry JA, Scriven GT (1965) Insectary production of phytoseiid mites. J Econ Entomol 58(2):282–284. 10.1093/jee/58.2.282

[CR22] McMurtry JA, Sourassou NF, Demite PR (2015) The Phytoseiidae (Acari: Mesostigmata) as biological control agents. In: Carrillo D, Moraes G, Peña J (eds) Prospects for biological control of plant feeding mites and other harmful organisms. Progress in Biological Control, v19. Springer, Cham. 10.1007/978-3-319-15042-0_5

[CR23] Michereff-Filho M, Navia D, Quevedo IA, Magalhães MA, Melo JWS, Lopes RB (2022) The effect of spider mite-pathogenic strains of *Beauveria bassiana* and humidity on the survival and feeding behavior of *Neoseiulus* predatory mite species. Biol Control 176:105083. 10.1016/j.biocontrol.2022.105083

[CR24] Mills N (2006) Interspecific competition among natural enemies and single versus multiple introductions in biological control. In: Brodeur J, Boivin G (eds) Trophic and guild interactions in biological control. Springer, Dordrecht, Netherlands, pp 191–220. 10.1007/1-4020-4767-3

[CR25] Palevsky E, Walzer A, Gal S, Schausberger P (2008) Evaluation of dry-adapted strains of the predatory mite *Neoseiulus californicus* for spider mite control on cucumber, strawberry and pepper. Exp Appl Acarol 45(1–2):15–27. 10.1007/s10493-008-9162-x 18566897

[CR26] Praslička J, Huszar J (2004) Influence of temperature and host plants on the development and fecundity of the spider mite *Tetranychus urticae* (Acarina: Tetranychidae). Plant Prot Sci 40(4):141–144. 10.17221/465-PPS

[CR27] Quesada-Moraga E, Garrido-Jurado I, Yousef-Yousef M, González-Mas N (2022) Multitrophic interactions of entomopathogenic fungi in biocontrol. Biocontrol 67:457–472. 10.1007/s10526-022-10163-5

[CR28] Rahmani H, Hoseini M, Saboori A, Walzer A (2016) Prey preference of the predatory mite *Neoseiulus californicus* (Mesostigmata: Phytoseiidae) when offered two major pest species, the two spotted spider mite and the onion thrips. Int J Acarol 42(6):319–323. 10.1080/01647954.2016.1191540

[CR29] Rioja C, Zhurov V, Bruinsma K, Grbic M, Grbic V (2017) Plant-herbivore interactions: a case of an extreme generalist, the two-spotted spider mite *Tetranychus urticae*. Mol Plant-Microbe Interact 30(12):935–945. 10.1094/MPMI-07-17-0168-CR 28857675

[CR30] Roy HE, Pell JK (2000) Interactions between entomopathogenic fungi and other natural enemies: implications for biological control. Biocontrol Sci Technol 10(6):737–752. 10.1080/09583150020011708

[CR31] SAS Institute (2002) The SAS System, Version 9.00. SAS Institute, Cary, NC

[CR32] Seiedy M, Saboori A, Allahyari H (2012) Interactions of two natural enemies of *Tetranychus urticae*, the fungal entomopathogen *Beauveria bassiana* and the predatory mite, *Phytoseiulus persimilis*. Biocontrol Sci Technol 22:873–882. 10.1080/09583157.2012.695010

[CR33] Shi WB, Zhang LL, Feng MG (2008) Field trials of four formulations of *Beauveria bassiana* and *Metarhizium anisoplae* for control of cotton spider mites (Acari: Tetranychidae) in the Tarim Basin of China. Biol Control 45(1):48–55. 10.1016/j.biocontrol.2007.11.006

[CR34] Souza-Pimentel GC, Reis PR, Liska GR, Cirillo MA (2017) Interspecific interaction between *Phytoseiulus macropilis* and *Neoseiulus californicus* (Acari: Phytoseiidae) preying on *Tetranychus urticae* (Acari: Tetranychidae) on rosebush growing. Int J Environ Agric Res 3(11):71–79. 10.25125/agriculture-journal-IJOEAR-NOV-2017-17

[CR35] Stark JD, Banks JE (2003) Population-level effects of pesticides and other toxicants on arthropods. Annu Rev Entomol 48:505–519. 10.1146/annurev.ento.48.091801.112621 12221038

[CR36] Ullah MS, Lim UT (2015) Laboratory bioassay of *Beauveria bassiana* against *Tetranychus urticae* (Acari: Tetranychidae) on leaf discs and potted bean plants. Exp Appl Acarol 65:307–318. 10.1007/s10493-014-9871-2 25500970

[CR37] Ullah MS, Lim UT (2017) Laboratory evaluation of the effect of *Beauveria bassiana* on the predatory mite *Phytoseiulus persimilis* (Acari: Phytoseiidae). J Invertebr Pathol 148:102–109. 10.1016/j.jip.2017.06.006 28629883

[CR38] Vacacela Ajila HE, Colares F, Lemos F, Marques PH, Franklin EC, Santos do Vale W, Oliveira EE, Venzon M, Pallini A (2019) Supplementary food for *Neoseiulus californicus* boosts biological control of *Tetranychus urticae* on strawberry. Pest Manag Sci 75(7):1986–1992. 10.1002/ps.5312 30610750

[CR39] Vacante V (2015) The Handbook of Mites of Economic Plants: Identification, Bioecology and Control. CABI International, Wallingford

[CR41] Vásquez C, Colmenárez YC, Greco N, Ramos M (2023) Current Status of Phytoseiid mites as biological control agents in Latin America and experiences from Argentina using *Neoseiulus californicus*. Neotrop Entomol 52(2):240–250. 10.1007/s13744-023-01026-4 PMC10997537 36811713

[CR40] Vázquez LL (2019) Interactions of entomopathogenic fungus with entomophagous insects in agroecosystems. In: Souza B, Vázquez L, Marucci R (eds) Natural enemies of insect pests in Neotropical agroecosystems. Springer, Cham. 10.1007/978-3-030-24733-1_14

[CR42] Vergel SJN, Bustos RA, Rodríguez CD, Cantor RF (2011) Laboratory and greenhouse evaluation of the entomopathogenic fungi and garlic– pepper extract on the predatory mites, *Phytoseiulus persimilis* and *Neoseiulus californicus* and their effect on the spider mite *Tetranychus urticae*. Biol Control 57:143–149. 10.1016/j.biocontrol.2011.02.007

[CR43] Walthall WK, Stark JD (1997) Comparison of two population-level ecotoxicological endpoints: the intrinsic (*rm*) and instantaneous (*ri*) rates of increase. Environ Toxicol Chem 16(5):1068–. 10.1002/etc.5620160529

[CR44] Weintraub P, Palevsky E (2008) Evaluation of an arid adapted strain of the predatory mites, *Neoseiulus californicus*, for spider mite control on greenhouse sweet pepper. Exp Appl Acarol 45(1–2):29–37. 10.1007/s10493-008-9169-3 18584132

[CR45] Wu SY, Gao YL, Zhang YP, Wang ED, Xu XN, Lei ZR (2014) An entomopathogenic strain of *Beauveria bassiana* against *Frankliniella occidentalis* with no detrimental effect on the predatory mite *Neoseiulus barkeri*: evidence from laboratory bioassay and scanning electron microscopic observation. PLoS ONE 9(1):e84732. 10.1371/journal.pone.0084732 PMC3891770 24454744

[CR46] Wu S, Xie H, Li M, Xu X, Lei Z (2016a) Highly virulent *Beauveria bassiana* strains against the two-spotted spider mite, *Tetranychus urticae*, show no pathogenicity against five phytoseiid mite species. Exp Appl Acarol 70:421–435. 10.1007/s10493-016-0090-x 27783179

[CR47] Wu S, Gao Y, Smagghe G, Xu X, Lei Z (2016b) Interactions between the entomopathogenic fungus *Beauveria bassiana* and the predatory mite *Neoseiulus barkeri* and biological control of their shared prey/host *Frankliniella occidentalis*. Biol Control 98:43–51. 10.1016/j.biocontrol.2016.04.001

[CR48] Wu S, HE Z, Wang E, Xu X, Lei Z (2017) Application of *Beauveria bassiana* and *Neoseiulus barkeri* for improved control of *Frankliniella occidentalis* in greenhouse cucumber. Crop Prot 96:83–87. 10.1016/j.cropro.2017.01.013

[CR49] Wu S, Xing Z, Sun W, Xu X, Meng R, Lei Z (2018) Effects of *Beauveria bassiana* on predation and behavior of the predatory mite *Phytoseiulus persimilis*. J Invertebr Pathol 153:51–56. 10.1016/j.jip.2018.02.014 29453965

